# A Morphological Study of the Meniscus, Cartilage and Subchondral Bone Following Closed-Joint Traumatic Impact to the Knee

**DOI:** 10.3389/fbioe.2022.835730

**Published:** 2022-03-21

**Authors:** T. L. Haut Donahue, G. E. Narez, M. Powers, L. M. Dejardin, F. Wei, R. C. Haut

**Affiliations:** ^1^ Department of Biomedical Engineering, University of Memphis, Memphis, TN, United States; ^2^ Department of Biomedical Engineering, University of Massachusetts Amherst, Amherst, MA, United States; ^3^ Department of Small Animal Clinical Sciences, Michigan State University, East Lansing, MI, United States; ^4^ Orthopaedic Biomechanics Laboratories, College of Osteopathic Medicine, Michigan State University, East Lansing, MI, United States

**Keywords:** impact injury, meniscus, cartilage, subchondral bone, knee

## Abstract

Post-traumatic osteoarthritis (PTOA) is a debilitating disease that is a result of a breakdown of knee joint tissues following traumatic impact. The interplay of how these tissues influence each other has received little attention because of complex interactions. This study was designed to correlate the degeneration of the menisci, cartilage and subchondral bone following an acute traumatic event that resulted in anterior cruciate ligament (ACL) and medial meniscus tears. We used a well-defined impact injury animal model that ruptures the ACL and tears the menisci. Subsequently, the knee joints underwent ACL reconstruction and morphological analyses were performed on the menisci, cartilage and subchondral bone at 1-, 3- and 6-months following injury. The results showed that the morphological scores of the medial and lateral menisci worsened with time, as did the tibial plateau and femoral condyle articular cartilage scores. The medial meniscus was significantly correlated to the medial tibial subchondral bone at 1 month (*p* = 0.01), and to the medial tibial cartilage at 3 months (*p* = 0.04). There was only one significant correlation in the lateral hemijoint, i.e., the lateral tibial cartilage to the lateral tibial subchondral bone at 6 months (*p* = 0.05). These data may suggest that, following trauma, the observed medial meniscal damage should be treated acutely by means other than a full or partial meniscectomy, since that procedure may have been the primary cause of degenerative changes in the underlying cartilage and subchondral bone. In addition to potentially treating meniscal damage differently, improvements could be made in optimizing treatment of acute knee trauma.

## Introduction

Menisci are fibrocartilaginous tissues in the knee joint that aid in load distribution and act to protect the underlying articular cartilage from excessive joint loads. Menisci are frequently torn either in isolated injuries or in combination with damage to the anterior cruciate ligament (ACL). It is well documented that following traumatic damage to the ACL and meniscus, 78–83% of patients will develop post-traumatic osteoarthritis (PTOA) 10–14 years post-injury, regardless of treatment modality (surgical reconstruction or conservative interventions) (Fink et al., 2001; von Porat et al., 2004). More recently, clinical studies have indicated that PTOA can develop as early as 3 years post-injury (Whittaker et al., 2015; Wang et al., 2020).

Knee PTOA is a disease that affects all tissues within the joint including menisci, articular cartilage, subchondral bone, synovium, fat pad and ligaments. The interplay of these tissues and how they affect each other is difficult to study and hence has received limited attention to date. In the Framingham OA study of 913 knees, meniscal damage in a given location was associated with higher regional tibial subchondral bone mineral density (BMD) (Lo et al., 2008). Human clinical studies have shown that there is a strong association between meniscal damage and cartilage loss (Hunter et al., 2006; Sharma et al., 2008), with less meniscal coverage and lower meniscal height increasing the risk of cartilage loss. Few studies, however, have analyzed all three major tissues in the joint, i.e., menisci, cartilage and subchondral bone, and investigated how the degree of degradation correlates with the development of osteoarthritis in the joint.

We have developed a closed-joint impact injury animal model that mimics the clinical development of PTOA following a traumatic insult. This model has been used to document the natural history of PTOA following closed-joint trauma (Fischenich et al., 2015; Pauly et al., 2015; Fischenich et al., 2017a). This model demonstrates degradative changes in the material properties of the menisci, articular cartilage and bone. These changes in our model include increased cartilage fissuring, decreased glycosaminoglycan content in both the cartilage and meniscus, as well as decreases in bone volume (BV) and BMD. However, the previous studies using our model did not attempt to correlate the degradation of the menisci, cartilage and subchondral bone to each other. More recently, our closed-joint impact injury model was combined with surgical reconstruction of the ACL (Narez et al., 2021a; Wei et al., 2021; Wei et al., 2022). Despite reconstruction of the ACL, decreases in the material properties of the menisci and the articular cartilage were still present, but minimal changes were documented in subchondral bone quality and morphometry. Again, there was no attempt to correlate the changes in the subchondral bone to those in the menisci or articular cartilage.

The goals of the current study were to document the degree of damage to menisci, cartilage and subchondral bone in a closed-joint impact injury animal model with ACL surgical reconstruction and determine if there was a potential correlation between the degree of damage in these joint tissues. It was hypothesized there would be a correlation between the level of meniscal degeneration with both the underlying cartilage and the subchondral bone within a given hemijoint.

## Methods

**FIGURE 1:**
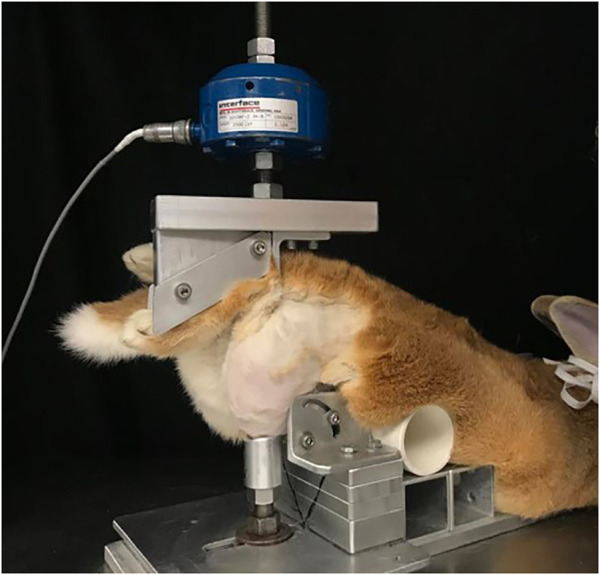
Injurious impact of rabbit knee set-up. In prone position (belly down) the rabbit’s hind paw was supported directly below the actuator of a servo-hydraulic machine. The knee was supported to prevent lateral and medial motion (with permission from Narez et al., 2021a).

All animal procedures for this study were approved by the Institutional Animal Care and Use Committee at Michigan State University (IACUC #05/16-073–00 and #PROTO201900255). Eighteen skeletally mature Flemish Giant rabbits (6.34 ± 0.75 kg, aged 6–9 months) were held in individual cages (32 × 32 × 28 in). The animals, a mix of male (*n* = 10) and female (*n* = 8), were anesthetized using standard protocols including 2% isoflurane in oxygen. The right hindlimb of each rabbit was shaved to expose the knee joint. The animals were then positioned into a rigid custom fixture in a servo-hydraulic testing system (Instron Corp, Norwood, MA) (Figure 1 **)** (Isaac et al., 2010a; Fischenich et al., 2015; Pauly et al., 2015; Fischenich et al., 2017a; Fischenich et al., 2017b; Narez et al., 2021a; Wei et al., 2021; Wei et al., 2022)**.** Using controlled impact with a 400 N preload, the actuator compressed the tibia downward 3.5 mm. This motion resulted in an anterior drawer of the knee where the tibia moved anteriorly with respect to the femur, causing ACL rupture. An auto-stop program was developed and used so that the test stopped automatically when there was an approximately 330 N load drop within 30 ms, which was indicative of a soft tissue (ACL) failure. ACL failure was confirmed by a board-certified veterinary surgeon (LMD) *via* a manual anterior drawer test of the joint. Force, time and displacement data were collected at 5 kHz. The left knee served as a contralateral control. We have previously shown that this impact injury animal model more closely mimics the clinical scenario following ACL rupture than the commonly used ACL surgical transection model, exhibiting many characteristics seen in human PTOA patients (Fischenich et al., 2017b). These animals were part of a much larger study with a total of 96 rabbits that investigated the efficacy of a surgical intervention and a pharmaceutical intervention in mitigating post-traumatic osteoarthritis (Narez et al., 2021a; Narez et al., 2021b; Wei et al., 2021; Narez et al., 2022; Wei et al., 2022). An animal model was necessary to recreate a true ACL/meniscus longitudinal injury study. Animals smaller than a rabbit often develop a calcified meniscus at an early age, hence the choice of the lapine model. The minimum number of animals was used such that a reasonable power (80%) was still obtained. No inhumane procedures were applied to the animals and they all experienced regular gait within 3 days after the intervention. Hence, this animal study fits the 3R’s principle (Hubrecht and Carter, 2019).

Approximately 2 weeks post-impact, a board-certified veterinary surgeon (LMD), performed a reconstruction of the torn ACL (Wei et al., 2021). Through a medial parapatellar arthrotomy, the condition of the ACL and meniscus was examined in more detail. If meniscal damage was evident, it was noted and treated with a partial or full meniscectomy. For a partial meniscectomy, only the injured or torn regions were removed. For extensive damage to the meniscus, a full meniscectomy was performed where the entire damaged medial meniscus was removed. Following meniscal debridement, to repair the torn ACL, the musculotendinous junction of the semitendinosus tendon (ST) was transected leaving the tibial attachment intact. At the ACL footprint, a 2.7 mm diameter tibial bone tunnel and a 3.2 mm diameter femoral tunnel were drilled. The ST free end was passed under the medial collateral ligament and through the tunnels via a suture loop. Tension was applied to the ST as it was secured to the femoral bone tunnel with a custom PEEK interference fit screw. To ensure the joint’s stability, post-reconstruction, an anterior drawer test was performed on the reconstructed knee. Postoperatively, the animals were monitored by a licensed veterinary nurse and randomly assigned to one of 3 groups. Rabbits were euthanized at 1-, 3- or 6-months post-trauma, with *n* = 6 in each time-point group. These timepoints were chosen to correspond to what others have done in the literature studying PTOA (Coleman et al., 2018; Lisee et al., 2021). Based on our previous studies using this animal model, and evaluation of mechanical properties of the cartilage and meniscus, an n = 6 in each group provides 80% power in the study (Narez et al., 2021a; Wei et al., 2022).

## Morphology Analysis

### Meniscus

**FIGURE 2:**
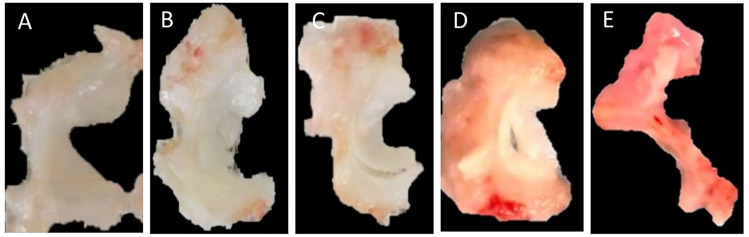
Morphological scoring of the meniscus. **(A)** 0 = normal, **(B)** 1 = surface fibrillation, **(C)** 2 = undisplaced tears, **(D)** 3 = displaced tears, **(E)** 4 = tissue maceration.

Following euthanasia, morphology grading of meniscal tissues was conducted by four separate blinded graders using a scoring system used by previous studies (Matyas et al., 2004; Pauli et al., 2011; Fischenich et al., 2014; Narez et al., 2021a). Each region of the meniscus (anterior, central and posterior) was scored. However, due to the fact that some knees had partial or full meniscectomies, regions were averaged to create one score for the lateral meniscus and one score for the medial meniscus. For both menisci, a score of 0 indicated normal tissue, a score of 1 indicated surface fibrillation, a score of 2 indicated un-displaced tears, a score of 3 indicated displaced tears, and a score of 4 indicated tissue maceration (Figure 2).

### Cartilage Morphological Assessment

**FIGURE 3:**
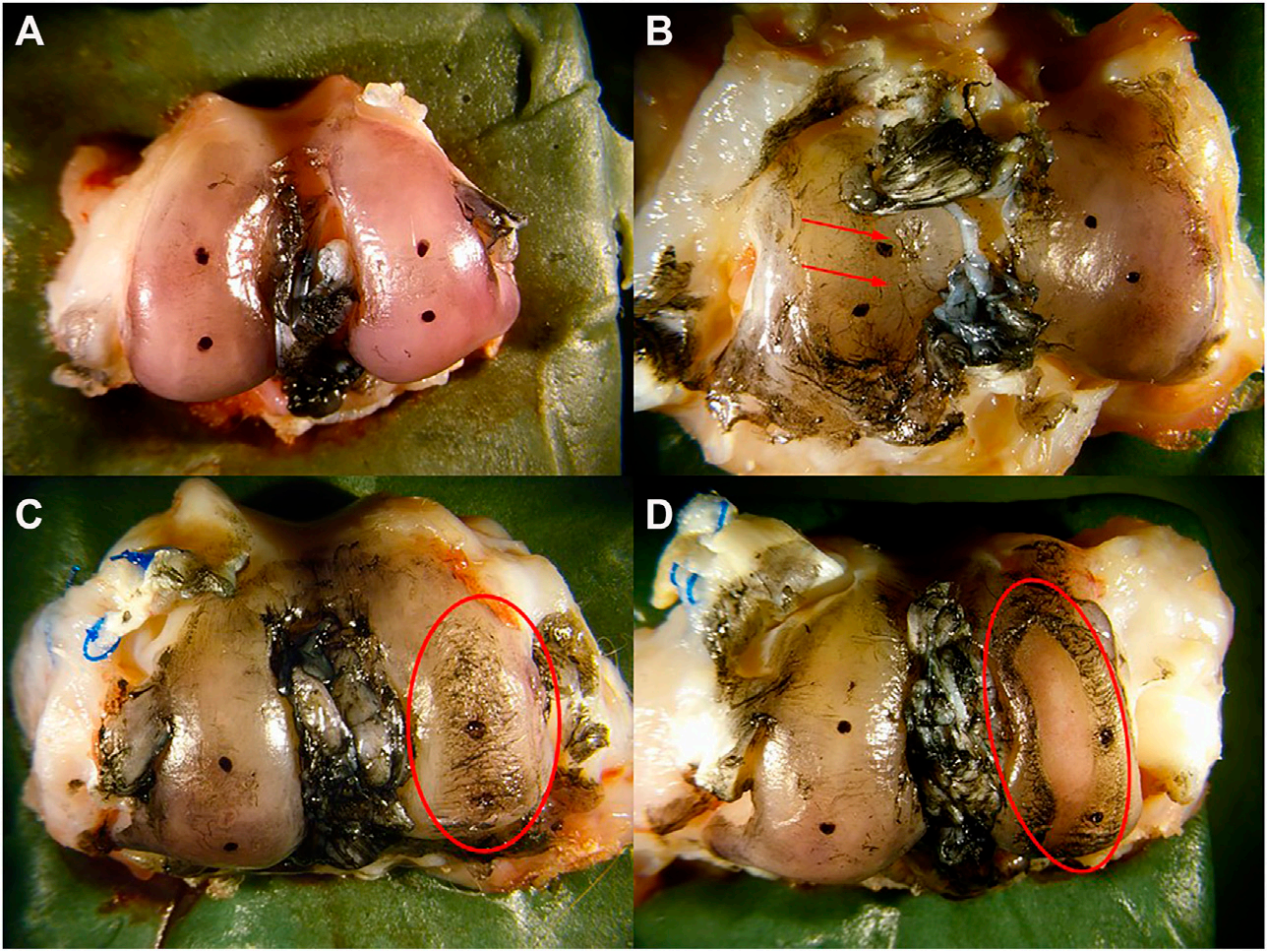
Morphological scoring of cartilage. **(A)** intact cartilage with the surface appearing normal was assigned a score of 1, **(B)** a few surface lesions (red arrows) that retain ink was assigned a score of 2, **(C)** moderate fibrillation retaining intense black patches of ink (red circle) was assigned a score of 3, and **(D)** full thickness erosion exposing underlying bone (red circle) was assigned a score of 4.

Morphology of the articular cartilage was assessed within 2 h following euthanasia. The surfaces of the tibial plateau and femoral condyles were stained with India ink to highlight surface fissures and other tissue irregularities. A blind morphological assessment (Yoshioka et al., 1996) was conducted by three independent graders with the following numerical grading scale: 1 = intact cartilage with the surface appearing normal, 2 = a few surface lesions that retained ink, 3 = moderate fibrillation retaining intense black patches of ink and 4 = full thickness erosion exposing underlying subchondral bone (Figure 3).

### Subchondral Bone

**FIGURE 4:**
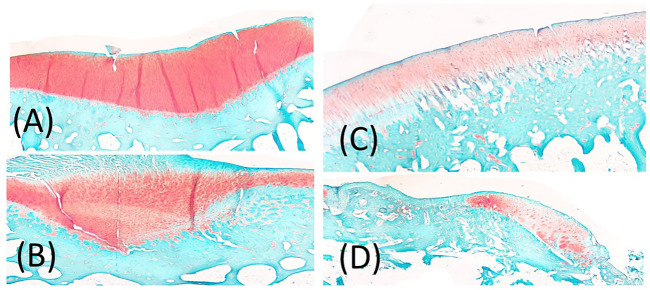
**(A)** representation of Score 1 = small spaces <50% of the length of condyle or plateau, **(B)** representation of 2 or 3 score depending on 2 = Moderate spaces/some splits <50% of the length, 3 = moderate spaces/splits >50% of the length, **(C)** 4 = numerous splits <50% of the length, **(D)** 5 = numerous splits >50% of the length.

Following euthanasia, bones were placed into a 10% neutral-buffered formalin solution. The limbs were decalcified in a 10% formic acid solution and sectioned to isolate regions of interest. The femoral condyles were sectioned along the parasagittal plane, passing through the medial and lateral condyles in a crani-caudal orientation, while the tibia was sectioned in the frontal plane, just anterior to the midline of the tibial plateau. Samples were processed, paraffin-embedded and sectioned at 7 μm. Sections were deparaffinized and stained with Hematoxylin, Saf-O and FG. Slides were imaged with a bright field microscope (Leica Microsystems Inc., Buffalo Grove, IL) and an Olympus DP25 camera (Olympus, Center Valley, PA). Three blind graders evaluated the subchondral bone morphology, using an OARSI score. Scores were reported and averaged before statistical analysis occurred. Scoring: 0 = normal, 1 = small spaces <50% of the length of condyle or plateau, 2 = Moderate spaces/some splits <50% of the length, 3 = moderate spaces/splits >50% of the length, 4 = numerous splits <50% of the length, 5 = numerous splits >50% of the length (Gerwin et al., 2010) (Figure 4).

### Statistical Analysis

Comparisons were only made within a hemijoint, not across hemijoints. Spearman Rank correlations were made between a) medial meniscus and medial cartilage of the tibial plateau, b) medial meniscus and medial cartilage of the femoral condyle, c) medial meniscus and medial tibial plateau subchondral bone, d) medial meniscus and medial femoral condyle subchondral bone, e) medial cartilage of the tibia and the medial tibial plateau subchondral bone, f) medial femoral cartilage and subchondral bone, g) lateral meniscus and lateral cartilage of the tibial plateau, h) lateral meniscus and lateral cartilage of the femoral condyle, i) lateral meniscus and the lateral tibial plateau subchondral bone, j) lateral meniscus and the lateral femoral condyle subchondral bone, k) lateral tibial plateau cartilage and subchondral bone, l) lateral femoral condyle cartilage and subchondral bone, m) lateral tibial cartilage and lateral femoral cartilage, and n) medial tibial cartilage and medial femoral cartilage. These correlations were made at 1, 3 and 6 months for a total of 42 correlations. Statistical significance was set as *p*-value <0.05.

## Results

All animals experienced ACL failure and were reconstructed. Of the 18 rabbits enrolled in the study, three rabbits had a full medial meniscectomy and 7 received a partial medial meniscectomy based on the level of damage observed in the medial meniscus. None of the animals had lateral meniscal damage that was noted during surgical reconstruction of the joint. The average morphological scores for the medial and lateral hemijoints, meniscus, femoral and tibial cartilage as well as tibial plateau and femoral condyle subchondral bone are presented in Table 1. Both the medial and lateral menisci tended to worsen with time, and the medial scores were higher than lateral scores. It is important to note that the lateral menisci had no indication of damage during reconstructive surgery, so the damage that developed to raise the scores over 6 months developed after reconstructive surgery. A similar trend was seen for the medial and lateral, tibial plateau and femoral condyle cartilage. Scores worsened with time, with 6 months scores being the highest. The subchondral bone of the tibial plateau and femoral condyles did not worsen with time and tended to show no consistent trends.

**TABLE 1:** Average morphological scores for all tissues studied.

	1 month	3 months	6 months
Medial Meniscus (scored 0–3)	2.19 ± 0.61	2.51 ± 0.45	2.49 ± 0.32
Lateral Meniscus (scored 0–3)	1.21 ± 0.27	2.04 ± 0.50	2.22 ± 0.29
Medial Tibial Plateau Cartilage (scored 1–4)	3.03 ± 0.17	3.77 ± 0.15	3.85 ± 0.15
Lateral Tibial Plateau Cartilage (scored 1–4)	3.18 ± 0.36	3.41 ± 0.35	3.79 ± 0.21
Medial Femoral Condyle Cartilage (scored 1–4)	2.93 ± 0.34	3.34 ± 0.34	3.53 ± 0.38
Lateral Femoral Condyle Cartilage (scored 1–4)	1.98 ± 0.33	2.07 ± 0.36	2.46 ± 0.36
Medial Tibial Plateau Subchondral Bone (scored 0–5)	1.50 ± 1.03	2.88 ± 1.56	2.13 ± 0.75
Lateral Tibial Plateau Subchondral Bone (scored 0–5)	2.25 ± 0.50	2.18 ± 1.14	1.43 ± 0.69
Medial Femoral Condyle Subchondral Bone (scored 0–5)	1.08 ± 0.30	2.79 ± 0.90	2.00 ± 1.78
Lateral Femoral Condyle Subchondral Bone (scored 0–5)	1.79 ± 0.51	1.46 ± 0.95	1.50 ± 0.64

Of the 42 possible correlations, only 4 were significant (Table 2). The medial meniscus was significantly correlated to the medial tibial subchondral bone at 1 month (*p* = 0.01) and the medial meniscus was significantly correlated to the medial tibial cartilage at 3 months (*p* = 0.04). There was only one significant correlation in the lateral hemijoint, the lateral tibial cartilage to the lateral tibial subchondral bone at 6 months (*p* = 0.05). The medial tibial cartilage significantly correlated to medial femoral cartilage at 6 months.

**TABLE 2:** Statistical values for all correlations between menisci, cartilage and subchondral bone for all comparisons at 1 month (mon), 3 and 6 months.

	Lateral meniscus	Medial meniscus	Lateral tibial cartilage	Medial tibial cartilage	Lateral femoral cartilage	Medial femoral cartilage
Lateral tibial cartilage	1 mon *p* = 0.12	N/A	N/A	N/A	See Other	N/A
	3 mon *p* = 0.09					
	6 mon *p* = 0.33					
Lateral femoral cartilage	1 mon *p* = 0.07	N/A	1 mon *p* = 0.21	N/A	N/A	N/A
	3 mon *p* = 0.39		3 mon *p* = 0.35			
	6 mon *p* = 0.11		6 mon *p* = 0.14			
Lateral tibial bone	1 mon *p* = 0.25	N/A	1 mon *p* = 0.20	N/A	N/A	N/A
	3 mon *p* = 0.21		3 mon *p* = 0.38			
	6 mon *p* = 0.09		**6** **mon *p* = 0.05**			
Lateral femoral bone	1 mon *p* = 0.36	N/A	N/A	N/A	1 mon *p* = 0.15	N/A
	3 mon *p* = 0.30				3 mon *p* = 0.15	
	6 mon *p* = 0.35				6 mon *p* = 0.10	
Medial tibial cartilage	N/A	1 mon *p* = 0.39	N/A	N/A	N/A	See Other
		**3** **mon *p* = 0.04**				
		6 mon *p* = 0.09				
Medial femoral cartilage	N/A	1 mon *p* = 0.10	N/A	1 mon *p* = 0.13	N/A	N/A
		3 mon *p* = 0.13		3 mon *p* = 0.48		
		6 mon *p* = 0.13		**6** **mon p = 0.02**		
Medial tibial bone	N/A	**1** **mon p = 0.01**	N/A	1 mon *p* = 0.38	N/A	N/A
		3 mon *p* = 0.21		3 mon *p* = 0.39		
		6 mon *p* = 0.38		6 mon *p* = 0.10		
Medial femoral bone	N/A	1 mon *p* = 0.19	N/A	N/A	N/A	1 mon *p* = 0.31
		3 mon *p* = 0.19				3 mon *p* = 0.28
		6 mon *p* = 0.33				6 mon *p* = 0.21

Bolded values represent significant correlations between row and column.

## Discussion

The study showed that significant correlations occurred between 1) the tibial plateau subchondral bone and cartilage in the lateral hemijoint, and 2) between the meniscus and tibial cartilage in the medial hemijoint, 3) between the meniscus and tibial subchondral bone in the medial hemijoint, and 4) between femoral and tibial cartilage in the medial hemijoint. There were no significant correlations between the femoral condyle cartilage and femoral subchondral bone. Hence, in the medial hemijoint, when the meniscus was damaged, the cartilage and subchondral bone were also significantly damaged as early as 1 month. This finding confirms that the menisci likely play a significant role in protecting the cartilage and bone (Fox et al., 2012; Abusara et al., 2018). However, in the lateral compartment, where no meniscal damage was noted during reconstructive surgery, no strong association between the meniscus and either the articular cartilage and underlying subchondral bone damage was noted. Interestingly, at later time points (6 months), the lateral tibial cartilage and tibial plateau bone were correlated. While there was no significant damage acutely to the lateral meniscus, morphological score worsened with time. We hypothesize that perhaps there was either a change in joint kinematics following reconstruction that affected the lateral compartment, or the medial meniscus damage changed the loading in the lateral hemijoint resulting in increased damage. Future studies will need to further investigate possible causes for this result.

Few studies have previously attempted to document correlations between the integrity of the three tissues studied here; meniscus, cartilage and subchondral bone. One previous study, using magnetic resonance imaging did show that meniscal extrusion predicts increases in subchondral bone lesions, expansion of subchondral bone and bone cysts, and is associated with decreases in tibial articular cartilage volume as well as increases in tibial plateau area (Wang et al., 2010). That study, however, did not report the degree of degeneration of the main body of the meniscus, only that it was extruded. Meniscal extrusion has also been associated with the development of osteophytes (Miller et al., 1997; Lerer et al., 2004). While these studies are interesting, they do not report the cause of meniscal extrusion, whether it be due to meniscal attachment laxity or degenerative changes in the main body of the meniscus.

Previous studies using the same animals as in this current study documented changes in the histological and biomechanical behavior of the cartilage, meniscus and bone (Narez et al., 2021a; Wei et al., 2022). Compared to their contralateral controls, reconstructed limbs showed osteoarthritic changes to both the lateral and the medial menisci, articular cartilage and subchondral bone as early as 1-month post-trauma. The degeneration progressed in all tissues over time up to 6-months. Overall, the medial compartments had more tissue damage than their corresponding lateral counterparts. The damage that was present in these rabbits mimics clinical observations of patients suffering ACL injury and undergoing reconstruction (Felson, 2004; Goldring and Goldring, 2016). There were overall decreases in the cartilage fiber modulus and matrix modulus, and an increase in the cartilage tissue permeability when comparing the reconstructed to the control limbs. Decreases in both the instantaneous and equilibrium modulus were documented in both menisci. Minimal changes were found in bone quality and morphometry; however, bones from the reconstructed limbs showed large volumes of osteophyte formations, with an increase in volume over time. (Narez et al., 2021a; Wei et al., 2022). The reconstructed limbs demonstrated increases in cartilage fissuring and subchondral bone spaces or splits, as well as decreases in cartilage glycosaminoglycan (GAG) staining and tidemark integrity, therefore generally exhibiting more tissue damage than their contralateral control limbs. The analyses of the subchondral bone thickness (SCBT) showed a general trend of thickening of the underlying bone following ACL injury and reconstruction at all three time-points (except for the lateral compartments of the femur and tibia at 6-months), with a significant increase of the SCBT in the medial compartment of the tibia at 6-months post-trauma. GAG coverage analyses found a significant decrease in coverage of the medial meniscus at the 1- month time point. In the lateral meniscus, there was a documented decrease in coverage at both 3 and 6-months. Cortical and trabecular microstructure worsened in the reconstructed limbs with time compared to contralateral limbs. These data indicate that reconstruction alone does not prevent osteoarthritic changes. Taken together with the data from this study correlating the cartilage, menisci and bone morphology, it is likely that there is a complex interplay amongst joint tissues and future work should investigate all joint tissues in a PTOA study and attempt to elude the starting point of damage to explore targeting early interventions to slow or prevent the onset and progression of PTOA.

The importance of subchondral bone in the development of OA has been well characterized in a review by (Li et al., 2013). This review documents the commonly seen subchondral bone sclerosis as a hallmark of OA in addition to microdamage, bone marrow edema-like lesions and bone cysts. Lacourt et al., 2012 studied the relationship between cartilage and subchondral bone in a racehorse model that is a natural model of repetitive trauma-induced osteoarthritis (Lacourt et al., 2012). While that study showed that cartilage damage was associated with bone degeneration, the meniscus was not investigated. In a guinea pig model of spontaneous OA, Zamli et al., 2014 showed that subchondral bone thickening preceded chondrocyte apoptosis and cartilage degradation (Zamli et al., 2014). Wang et al., 2013 showed that subchondral bone changes (i.e., increased thickness, bone mineral density and a decrease in porosity) occurred prior to cartilage degeneration (Wang et al., 2013). While these studies suggest that subchondral bone changes precede cartilage changes in spontaneous OA (Wang et al., 2013; Zamli et al., 2014), which tissue is first affected and potentially induces pathological changes in other joint structures after acute trauma has yet to be elucidated. If the order or sequence of degeneration were known, better therapeutic interventions could be investigated, improving the outcome of ACL replacement surgery or joint treatment following a traumatic impact event.

The current study strongly suggested that 1) early meniscal damage in the medial hemijoint resulted in subsequent damage to the medial cartilage and underlying tibial subchondral bone and 2) early medial meniscal damage likely affected the lateral hemijoint. Our model delivered a single injurious impact to the knee joint that resulted in ACL failure and either no or partial medial meniscal damage. Based on observable structural damage, partial or full medial meniscectomy was performed at the time of ACL reconstructive surgery. Degenerative changes in the medial meniscus, femoral and tibial articular cartilage and subchondral bone were correlated in a first attempt to help understand potential relationships between these three joint tissues after a single blunt joint impact. The study was limited by the fact that not all animals had meniscal damage requiring a meniscectomy and by the relatively small sample size (*n* = 6) in each group. Another limitation is that we did not apply a fixed pre-tension to the ACL reconstruction in the current study. However, clinically a fixed pre-tension is not used, and further, the pre-tension of the native tissue is not known, so what pre-tension to use remains unclear. Despite these limitations, these data suggested that, following trauma, the observed medial meniscal damage should be treated acutely by means other than a full or partial meniscectomy, since that procedure may have been the primary cause of degenerative changes in the underlying cartilage and subchondral bone. In fact, a clinical study confirms that repairs of meniscal damage are more favorable than meniscectomies (Lutz et al., 2015). Meniscal repair and substitution are becoming more commonplace and future research should continue to optimize these techniques (Paxton et al., 2011; Zaffagnini et al., 2011). Additionally, perhaps other improvements that aim to optimize acute knee trauma should be explored, such as treatment with Poloxamer 188 (P188) which acts to reseal cell membranes following traumatic rupture (Serbest et al., 2006; Natoli and Athanasiou, 2008; PascualGarrido et al., 2009; Bajaj et al., 2010; Isaac et al., 2010b; Coatney et al., 2015; Narez et al., 2021b). And yet, the current study also showed degenerative changes in the lateral cartilage and underlying bone without early observed damage to the overlying lateral meniscus post-trauma. Thus, while PTOA in the joint was likely induced by structural tissue changes in the medial hemijoint, there were likely other pathological processes at play.

## Data Availability

The original contributions presented in the study are included in the article/Supplementary Material, further inquiries can be directed to the corresponding author.
